# Peak Performance: Advances in Macromolecular MicroED Data Collection

**DOI:** 10.1063/4.0000996

**Published:** 2025-10-27

**Authors:** Max T. B. Clabbers

**Affiliations:** Interdisciplinary Nanoscience Center, Aarhus University, Aarhus, Mid-Jutland, Denmark. Department of Molecular Biology and Genetics, Aarhus, Mid-Jutland, Denmark

## Abstract

High-resolution data and accurate intensity measurements are crucial for resolving detailed structural models in macromolecular electron crystallography. However, obtaining high-quality structural information at atomic resolution is generally difficult, especially for crystalline biological specimens, as the diffracted intensities rapidly fade at higher resolution. Increasing the fluence does not circumvent this problem, as it results in detrimental radiation damage that compromises data quality. The diffraction signal at low flux density conditions is therefore generally expected to be relatively weak, limiting the attainable resolution. Recent advances in macromolecular MicroED data collection have substantially improved data quality, making it possible to extract high-resolution information from small nanocrystals that would otherwise be difficult to analyze. Notably, the introduction of hybrid pixel detectors and direct electron detectors has revolutionized diffraction data acquisition. These cameras essentially have no read-out noise and greatly improve the signal-to-noise ratio, which is particularly beneficial for recording reliable data from faint high- resolution reflections. In many cases, data quality and resolution can further be improved using energy filtering, which decreases background noise and sharpens diffraction peaks by removing inelastically scattered electrons. These technological advances allow for improved data collection strategies, enabling more accurate intensity measurements at higher resolution. Better data leads to better structural models that provide deeper insights into protein structure and function.

